# Reproductive Transitions and Sperm Utilisation in a Facultatively Parthenogenetic Stick Insect

**DOI:** 10.1002/ece3.71766

**Published:** 2025-07-07

**Authors:** Jigmidmaa Boldbaatar, Pietro Pollo, Daniela Wilner, Nathan W. Burke, Russell Bonduriansky

**Affiliations:** ^1^ Evolution and Ecology Research Centre, School of Biological, Earth and Environmental Sciences University of New South Wales Sydney New South Wales Australia; ^2^ School of Environmental and Life Sciences University of Newcastle Callaghan New South Wales Australia; ^3^ Institute of Cell and Systems Biology of Animals Universität Hamburg Hamburg Germany

**Keywords:** fertilisation success, phasmid, reproductive switch, sperm precedence

## Abstract

Facultative parthenogenesis enables females to switch from asexual (parthenogenetic) to sexual reproduction after mating, but the process of fertilisation is poorly understood in such animals. In particular, it is not known whether switching reproductive modes requires changes in the eggs themselves, delaying the transition from laying unfertilised to fertilised eggs. Likewise, very little is known about patterns of sperm precedence in facultatively parthenogenetic females that mate with multiple males. In this study, we manipulated reproductive mode in females of the facultatively parthenogenetic stick insect *Megacrania batesii*. We used offspring sex ratio, fertilisation rate and paternity analysis to investigate how females descended from distinct natural populations switch between reproductive modes and utilise sperm from different males. In the switch treatment group, females were first allowed to lay unfertilised eggs and then paired with a male. In the non‐switch treatment group, females were instead paired successively with two different males. We collected eggs laid over two successive 10‐day periods after male introduction (switch treatment) or substitution (non‐switch treatment). We found little difference between the treatment groups in fertilisation rate or in the number of sons produced during the first and second 10‐day egg collection. We also observed similar reproductive performance between switch and non‐switch treatment groups, but females' population of origin influenced fertilisation rate and offspring sex ratio. In the non‐switch group, we found near‐equal fertilisation rates by the first and second male. Our results show that *M. batesii* females can quickly switch from producing parthenogenetic to fertilised eggs, suggesting that this transition does not require production of distinct types of eggs. Our results also show that *M. batesii* females can rapidly utilise sperm from a new mate and exhibit near‐complete sperm mixing, which suggests that paternity may be evenly distributed in this species.

## Graphical abstract

Facultative parthenogenesis enables females to switch from asexual (parthenogenetic) to sexual reproduction after mating, but the process of fertilisation is poorly understood in such animals. It is unknown whether switching reproductive modes requires changes in the eggs themselves, delaying the transition from laying unfertilised to fertilised eggs. Likewise, very little is known about patterns of sperm precedence in facultatively parthenogenetic females that mate with multiple males.

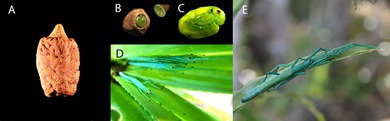

## 1 Introduction

Facultative parthenogenesis is a unique reproductive system that enables females to switch from asexual (parthenogenetic) to sexual reproduction following mating (Bedford 1978; Galis and van Alphen 2020; Simon et al. 2003). As a result of this switch, facultatively parthenogenetic (thelytokous) females can transition from laying unfertilised eggs (which typically produce only female offspring) to fertilised eggs (which produce both male and female offspring) (Bedford 1978; Suomalainen 1950). Many facultatively parthenogenetic species also show spatial sex ratio variation, with some populations consisting of females only (all‐female) and other populations consisting of both sexes (mixed‐sex) (Buckley et al. 2009; Miller et al. 2024; Morgan‐Richards et al. 2010; Wilner, Boldbaatar, et al. 2025). Facultatively parthenogenetic animals can provide valuable insights on the evolution of sexual and asexual reproductive modes (Burke and Bonduriansky 2017; Williams 1975), as well as on the role of sexual conflict in the maintenance of sexual reproduction (Burke and Bonduriansky 2019). However, many aspects of the reproductive biology of such species are still poorly known.

Several studies have modelled and reviewed the potential costs and benefits of sexual and asexual reproduction (Burke and Bonduriansky 2018b; Green and Noakes 1995; Hurst and Peck 1996; Lehtonen et al. 2012) and suggest that sexual conflict may be elevated in facultatively parthenogenetic species (Burke and Bonduriansky 2017); empirical work is rare but largely consistent with this prediction. For instance, in the stick insect *Extatosoma tiaratum*, switching from asexual to sexual reproduction appears to be costly for females, potentially selecting for strategies to avoid mating or fertilisation after the onset of parthenogenetic reproduction (Burke et al. 2015). A delay in switching could also result from physiological constraints (Engelstädter 2008; Schwander et al. 2013). For example, switching from asexual to sexual reproduction might require changes in gene expression in the female reproductive system (Schwander et al. 2013) and a physiological transition from the production of eggs that undergo automixis (i.e., fusion of the ovule with another meiotic product to restore diploidy) to the production of eggs that require fertilisation to begin development (Engelstädter 2017). Moreover, selection might favour adjustments to oogenesis depending on whether or not the eggs are likely to be fertilised because of differences in offspring sex ratio, such as production of males exclusively or primarily from fertilised eggs (thelytoky) or unfertilised eggs (arrhenotoky). Male and female embryos may have somewhat different requirements, selecting for changes in egg provisioning.

Females' propensity to switch between reproductive modes could also vary across populations. Previous research on facultatively parthenogenetic stick insects has shown that males have poor fertilisation success when paired with females descended from all‐female populations, compared to females descended from mixed‐sex populations (Morgan‐Richards et al. 2019; Wilner, Boldbaatar, et al. 2025). It is possible that females from all‐female populations, where mating almost never occurs and reproduction is solely asexual, have lost the ability to quickly switch to sexual reproduction when mated due to increased selection for effective parthenogenesis and relaxed selection on the traits that enable sexual reproduction (van der Kooi and Schwander 2014).

When mating and fertilisation do occur, females can be choosy and employ cryptic mate choice mechanisms that bias fertilisation rates by sperm provided by different males (Eberhard 1991; Kokko et al. 2003). Likewise, male adaptations (i.e., physical, behavioural and physiological traits) can enhance performance in sperm competition (Parker 2020; Stockley 1997). In many insects that encounter multiple mating partners, the order of male partners plays an important role in determining whose sperm is more likely to fertilise eggs (i.e., sperm precedence) (Parker 1970b). Eggs can be predominantly fertilised by the first male (first‐male sperm precedence; Marshall et al. 2004), the last male (last‐male sperm precedence; Mclain 1989) or both males in equal proportion (sperm mixing; Zeh and Zeh 1994). Understanding sperm precedence could provide insights into dynamics of sexual and asexual reproduction, as well as switching strategies in facultative parthenogenesis. If females employ an ‘honest raffle’ system that allows for fertilisation by stored sperm from multiple males (Parker and Pizzari 2010), that would suggest a tendency to diversify their offspring's genetic makeup. Such a strategy could be particularly advantageous in populations facing challenging environmental conditions, as genetic diversity enhances adaptability (Cornell and Tregenza 2007). Alternatively, if sperm mixing or displacement (resulting in last‐male sperm precedence) occurs, sexual selection can favour coercive mating behaviour to enable take‐overs, and prolonged mate guarding by males as a male strategy to mitigate sperm competition risk (Parker 1970a; Storey et al. 1995), which could impose additional costs on females (Jormalainen and Merilaita 1995). To our knowledge, nothing is known about patterns of sperm precedence in facultatively parthenogenetic insects. Likewise, it is unknown whether facultatively parthenogenetic females show selectivity or control over fertilisation (Walker 1980).

The peppermint stick insect (*Megacrania batesii*) is a facultatively parthenogenetic (thelytokous) species in which every female is capable of reproducing both asexually and sexually. The mechanism of parthenogenesis in this species is either gamete duplication or terminal fusion with no recombination (Miller 2025). Stick insects typically have an XX/XO or XX/XY sex determination system (Blackmon et al. 2017), but the sexual karyotype of *M. batesii* is not yet known. An unmated female that has already started laying parthenogenetic eggs can switch from asexual to sexual reproduction if mating and fertilisation occur, and offspring from unfertilised eggs can also continue to be produced after mating (Miller 2025; Wilner, Boldbaatar, et al. 2025). *M. batesii* females produce an even sex ratio from fertilised eggs, but exclusively female offspring from unfertilised eggs (Wilner, Boldbaatar, et al. 2025). Natural populations of *M. batesii* exhibit sex ratio variation. Some are all‐female populations, where reproduction is exclusively asexual, and others are mixed‐sex, where reproduction is mostly sexual (Miller et al. 2024). These populations also exhibit two distinct genetic clusters (‘Northern’ vs. ‘Southern’ genotypes) in far‐north Queensland, Australia (Miller et al. 2024). Most known Southern‐genotype populations are all‐female (Miller et al. 2024), and females from these populations exhibit evolved resistance to fertilisation (Wilner, Boldbaatar, et al. 2025). Conversely, most known Northern‐genotype populations are mixed‐sex, and females from these populations show little or no resistance to fertilisation. Interestingly, as previously reported for *E. tiaratum* (Burke and Bonduriansky 2022), asexually produced females from mixed‐sex *M. batesii* populations also show partial resistance to mating and/or fertilisation (Wilner, Boldbaatar, et al. 2025). In mixed‐sex populations, males engage in prolonged mate guarding (often lasting for a couple of weeks based on field observations), and both sexes can mate with multiple partners (Boldbaatar et al. 2024). This indicates that females may receive sperm from multiple males throughout their lifespan, potentially causing sperm competition. However, it is unknown what the pattern of sperm precedence is in *M. batesii* or how quickly females can switch from using the sperm of the initial male to that of a subsequent mate.

Here, we carried out a laboratory experiment on *M. batesii* females to investigate (1) how quickly females switch from asexual to sexual reproduction, (2) whether the speed of switching between reproductive modes depends on the population of origin (Southern all‐female populations vs. Northern mixed‐sex populations), (3) the effect of reproductive mode switches on reproductive performance and (4) the sperm precedence pattern in polyandrous females. If the switch from asexual to sexual reproduction does not require the production of different eggs in response to male cues, then male pairing should result in a rapid switch to production of fertilised eggs. By contrast, if switching between distinct reproductive modes requires an adjustment in the eggs themselves (e.g., using different mechanisms of oogenesis to produce haploid or diploid eggs, or adjustments to egg provisioning), a delay in the onset of fertilisation is expected because *M. batesii* eggs take several days to mature inside the ovaries and female reproductive tract (Vasconcelos et al. 2025). We also asked whether females descended from Southern all‐female populations, which are resistant to fertilisation, exhibit a greater delay in switching from laying unfertilised to fertilised eggs than do females from Northern mixed‐sex populations. Likewise, we asked whether a reproductive switch decreases reproductive performance as a result of costly physiological adjustments. Because *M. batesii* males guard females (Boldbaatar et al. 2024), we predicted that last‐male sperm precedence or sperm mixing occurs in this species.

## 2 Materials and Methods

### 2.1 Insect Maintenance and Experimental Design

**FIGURE 1:**
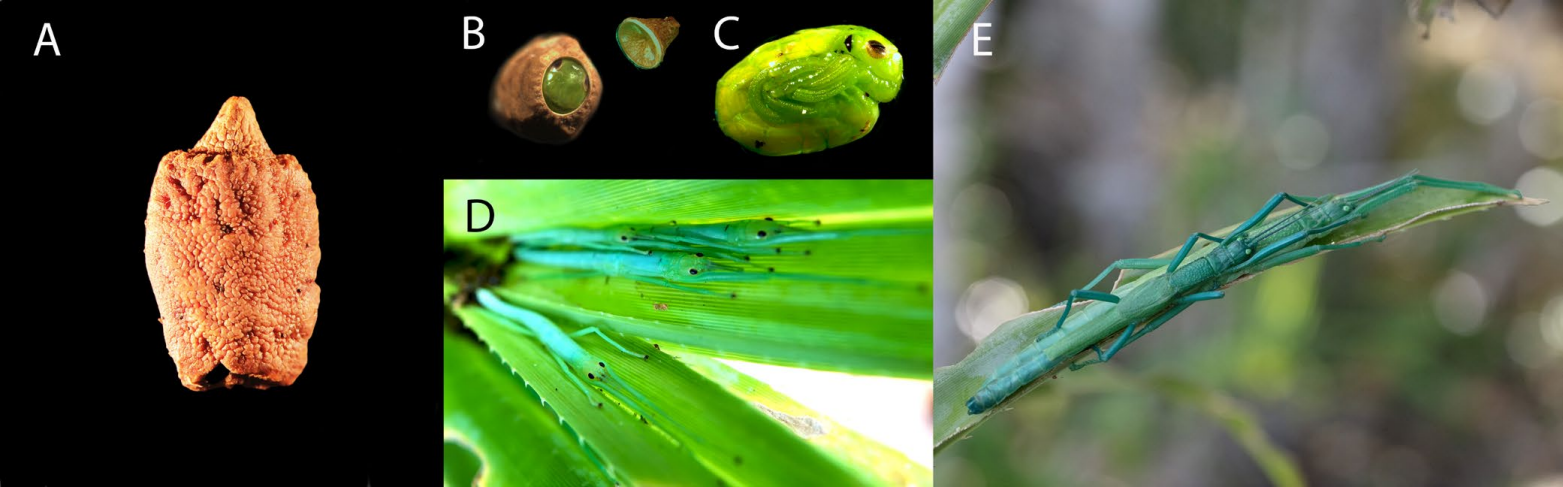
(A) *Megacrania batesii* egg; (B) hatchling ready to exit the egg through the opening formed by removing the egg cap (top right); (C) mature embryo; (D) hatchlings on *Pandanus tectorius* leaves; (E) adult female with guarding male on a *Pandanus tectorius* leaf. Photo credit: Russell Bonduriansky.

We used lab‐reared females that were collected as eggs from natural Northern genotype mixed‐sex (BK, CO, MB and MK) and Southern genotype all‐female (B1, CB and TB) populations (Figure 1) of *M. batesii* in far‐north Queensland, Australia (population codes correspond to Wilner, Boldbaatar, et al. 2025). This laboratory study was conducted between the years 2020 and 2021. All individuals were kept at 25°C ± 2°C and 60% ± 10% humidity. Each adult was kept individually in a cylindrical container (20 × 40 cm), fed *Pandanus tectorius* leaves and watered daily prior to the experiment. After their adult moult, *M. batesii* females developed for an average of 23.7 days (standard deviation = 3.25 days) before starting to lay eggs, and the duration of this pre‐oviposition phase was not affected by male presence, population type or their interaction (ANOVA: all *F* < 1.5, all *p* > 0.2).

**FIGURE 2:**
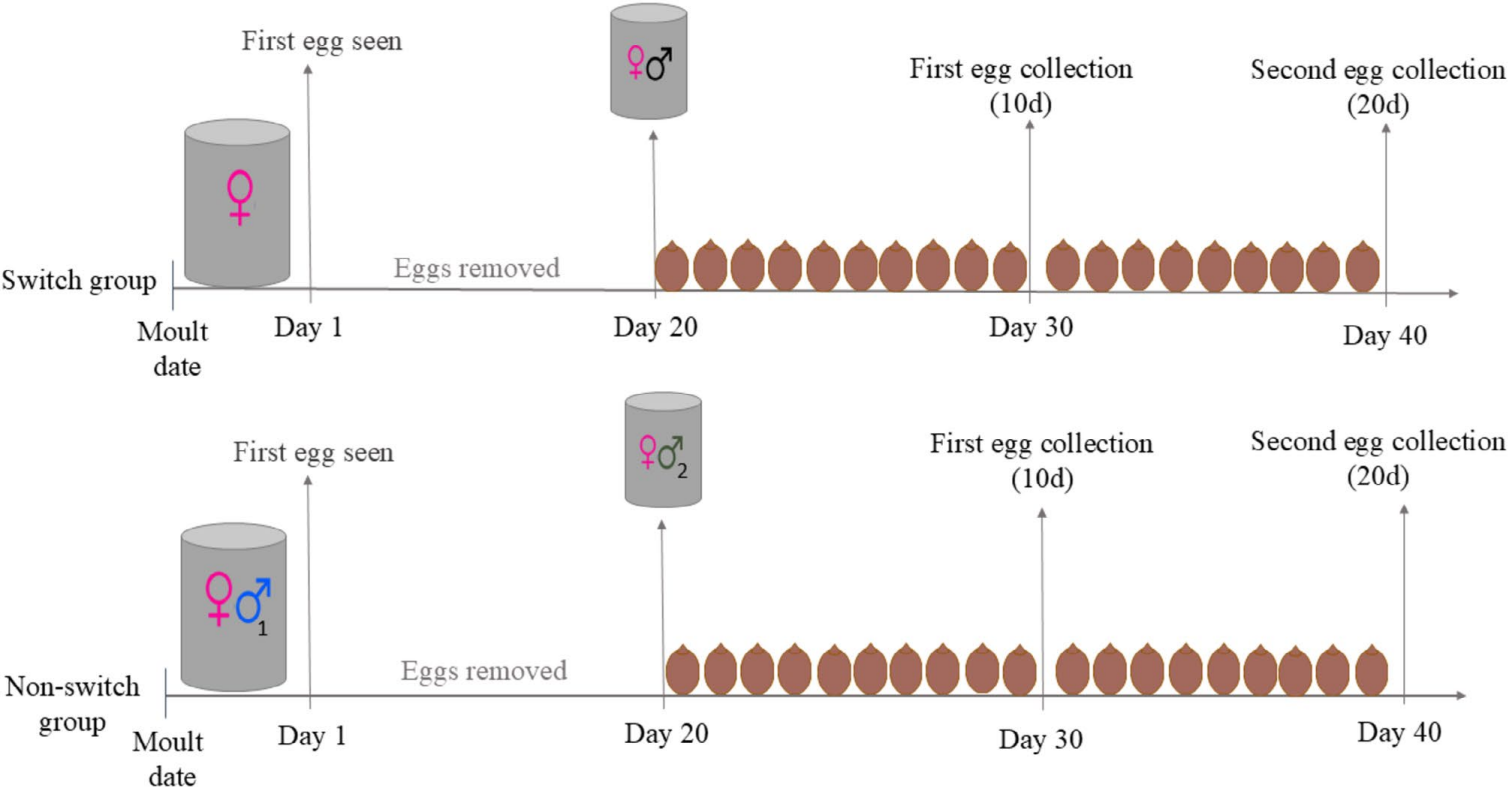
Experimental design. In the switch group, females were housed individually and, after the onset of oviposition (Day 1), were allowed to lay eggs for 20 days. All eggs were then removed, each female was paired with a male for a further 20 days, and eggs laid during this period were collected in two batches (at Days 30 and 40). In the non‐switch group, females were housed with a male (first male) and, after the onset of oviposition (Day 1), allowed to mate and lay eggs for 20 days. All eggs were then removed, each female was paired with a different male (second male) for a further 20 days, and eggs laid during this period were collected in two batches (at Days 30 and 40). Note that the number of eggs shown is for illustrative purposes and does not represent the actual count.

Adult females were divided into two treatment groups: ‘switch’ (*N* = 17) and ‘non‐switch’ (*N* = 18). In the switch group, females were first housed alone and, after the onset of oviposition, allowed to lay unfertilised eggs for 20 days. In the non‐switch group, females were paired with a male (first male) soon after their adult moult (mean = 1.78 ± 1.06 days) and, after the onset of oviposition, allowed to mate and lay eggs for 20 days. The eggs laid over the first 20 days were then removed. The number of eggs laid during this 20‐day period did not differ between unpaired (switch treatment) females and male‐paired (non‐switch treatment) females (see figure 4A in Wilner, Boldbaatar, et al. 2025). Switch treatment females were then paired with a male for 20 days, whereas non‐switch treatment females were paired with a new male (second male) for 20 days (Figure 2). Following male‐pairing (switch treatment) or replacement (non‐switch treatment), we collected eggs laid by each female over two successive 10‐day periods. Eggs from each female were kept in 125‐mL containers with perforated lids and moist cocopeat until hatching. The hatchlings from these eggs were checked and sexed daily based on abdominal sternite morphology (Miller et al. 2024), and then frozen at −80°C for DNA sequencing. Both treatment groups contained a similar number of individuals from Northern mixed‐sex and Southern all‐female populations. However, as these two types of populations differ both in genotype and in typical mode of reproduction (Miller et al. 2024), we cannot decouple these factors in this study.

We quantified hatchling sex and heterozygosity (see details below) to estimate fertilisation rates across the two egg collection periods and thus to determine the rate of reproductive mode switches. We also quantified fecundity (number of eggs laid), egg development time (number of days from the beginning of each 10‐day egg collection period to hatching) and egg hatching success (proportion of eggs that hatched in each brood) to determine whether switching imposes a reproductive cost. Finally, we carried out paternity analysis (see below) to investigate patterns of sperm use in offspring from the non‐switch treatment group.

### 2.2 DNA Extraction and Sequencing

All DNA isolations were performed using the Gentra Puregene kit from Qiagen by following the manufacturer's protocol (DNA purification from Mouse Tail Tissue Using the Gentra Puregene Mouse Tail Kit). The protocol was slightly modified for *M. batesii* (see details in Miller et al. 2024). All isolated DNA samples were submitted to Diversity Arrays Technology (Canberra, Australia) for sequencing using the DArTAG protocol, which analyses a subset of variable loci across the genome. Genotypes were obtained for 300 single‐nucleotide polymorphism (SNP) loci, of which 260 loci yielded high‐quality sequences (Miller 2025). In total, we sequenced 208 female hatchlings (hereafter daughters) from 30 families (17 families from the non‐switch; 13 families from the switch group), and 45 male hatchlings (hereafter sons) from seven families from the non‐switch group.

### 2.3 Determination of Fertilisation Rate and Paternity

In *M. batesii* , asexual reproduction typically yields individuals with much lower heterozygosity compared to sexual reproduction (Miller et al. 2024; Wilner, Boldbaatar, et al. 2025), and heterozygosity can therefore be used to determine whether individuals have developed from fertilised or unfertilised eggs. In previous studies based on DArTAG sequencing, asexually produced individuals, including field‐collected eggs from all‐female populations and eggs from unmated females in the lab, typically showed heterozygosity values of 0.06 or below, while eggs laid by mated females showed heterozygosity values greater than 0.14 (Miller et al. 2024; Wilner, Boldbaatar, et al. 2025). We therefore set a threshold of heterozygosity greater than 0.06 as indicative of development from a fertilised egg. Previous studies showed that a small fraction of parthenogenetically produced individuals retained higher heterozygosity values (Miller 2025), but these values were generally below the range of heterozygosities observed in sexually produced individuals from the populations (Miller 2025) used in the present study. A strongly bimodal distribution of heterozygosity values was observed in our study (Figure S1) and nearly all sequenced individuals showed a strong genetic match to one of the potential sires (see below), supporting the use of heterozygosity to distinguish sexually versus parthenogenetically produced females.

Based on the sequenced daughters' heterozygosity values, we found 94 daughters developed from fertilised eggs (from 20 families: non‐switch = 12; switch = 8) and 114 daughters developed from unfertilised eggs (from 27 families: non‐switch = 14; switch = 13). The total number of sequenced daughters (*N* = 208) represents 43.4% of hatched daughters, as another 271 daughters were not sequenced (Figure S1). To estimate the fertilisation proportion of all offspring per brood (i.e., sequenced and non‐sequenced individuals), we extrapolated based on the fertilisation proportion of sequenced daughters. However, for this extrapolation, we excluded broods with < 4 sequenced daughters within each egg collection for each mother, since estimates of fertilisation rate from small samples are imprecise. Doing so reduced the sample size of families to 20 (non‐switch = 10; switch = 10), the number of sequenced daughters to 185 and the number of non‐sequenced daughters to 111. Then, accounting for the number of sons (*N* = 92, all of which developed from fertilised eggs) hatched from both switch and non‐switch groups, we calculated the total fertilisation proportion of the offspring in each brood as (estimated number of daughters produced from fertilised eggs + total number of sons)/total number of offspring.

We performed paternity analysis on eight families from the non‐switch treatment group, in which females were mated with two males sequentially. The eight families were selected based on the availability of the parents' DNA sequence data. In those eight families, we used 40 fertilised daughters and 44 sequenced sons in total to assign their potential sire. From each of these families (where possible), we used genotypes at the 260 SNP loci from the mother, the two potential sires and their male and female offspring. Paternity was assigned to each offspring based on allelic mismatch between the offspring and the first and second males that the female had been paired with. Specifically, we tallied the number of loci for which the offspring genotype did not match the genotype of each of the two males (i.e., could not have resulted from a mating between the known mother and that male) and designated the male with the fewest mismatched loci as the sire. This yielded a clear pattern, where almost all sequenced offspring had a much smaller number of mismatched loci with one of the males than with the other (Figure S3). For 18 offspring from two brood families, we were only able to obtain a DNA sequence from one of the two potential sires. Therefore, for these 18 offspring, paternity was based on the number of mismatched loci between the genotyped male and the offspring. Based on data from the other sequenced offspring (Figure S3), we designated the sequenced male as the sire if the number of mismatched loci was < 20 and the other male as the sire if the number of mismatched loci was 20 or more.

### 2.4 Statistical Analysis

We fitted generalised linear mixed models (GLMMs) with a binomial error distribution for the proportion of offspring fertilised, the proportion of male offspring, the proportion of hatched eggs and the proportion of offspring sired by the first male in the non‐switch treatment group. We fitted a GLMM with a Poisson error distribution for fecundity (i.e., number of eggs laid), and a Gaussian linear mixed model (LMM) for egg development time. Almost all models included the same fixed factors: treatment (switch vs. non‐switch treatment as a categorical variable), female population type (Northern mixed‐sex vs. Southern all‐female as a categorical variable), egg collection period (first 10‐day egg collection period vs. second 10‐day egg collection period as a categorical variable) and all two‐ and three‐way interactions. The only exception to this was the GLMM exploring paternity rates, which did not include treatment as a fixed factor because it only used data from the non‐switch treatment. Female ID was included as a random effect in all models. We initially also included offspring sex (and its interactions with other predictor variables) as a fixed factor in the development time LMM (see full model details in Table S1), but the inclusion of this variable was not supported based on a likelihood ratio test when compared with a reduced model without offspring sex (*χ* ^2^ = 13.61, df = 8, *p* = 0.092), and the development time model was therefore refitted without offspring sex.

We carried out all statistical analyses in R version 4.4.3 (R Core Team 2025). We fitted GLMMs with the function *glmmTMB* from the package *glmmTMB* (Brooks et al. 2017), and the LMM with the *lmer* function from the package *lme4* (Bates et al. 2007). GLMM effects were tested using Wald *z*‐tests. We made ad hoc pairwise comparisons using the function *emmeans* from the package *emmeans* (Lenth 2025). We also examined whether paternity success differed overall between the first and second male using a Wilcoxon matched‐pairs test, with female identity as the grouping factor. Model assumptions were verified using the package DHARMa (Hartig 2024). We did not detect overdispersion in any of the models according to the function *check_overdispersion* from the package *performance* (Lüdecke et al. 2021). Unless stated otherwise, estimates reported in the manuscript represent mean ± standard deviation.

## 3 Results

### 3.1 Fertilisation Rate

**FIGURE 3:**
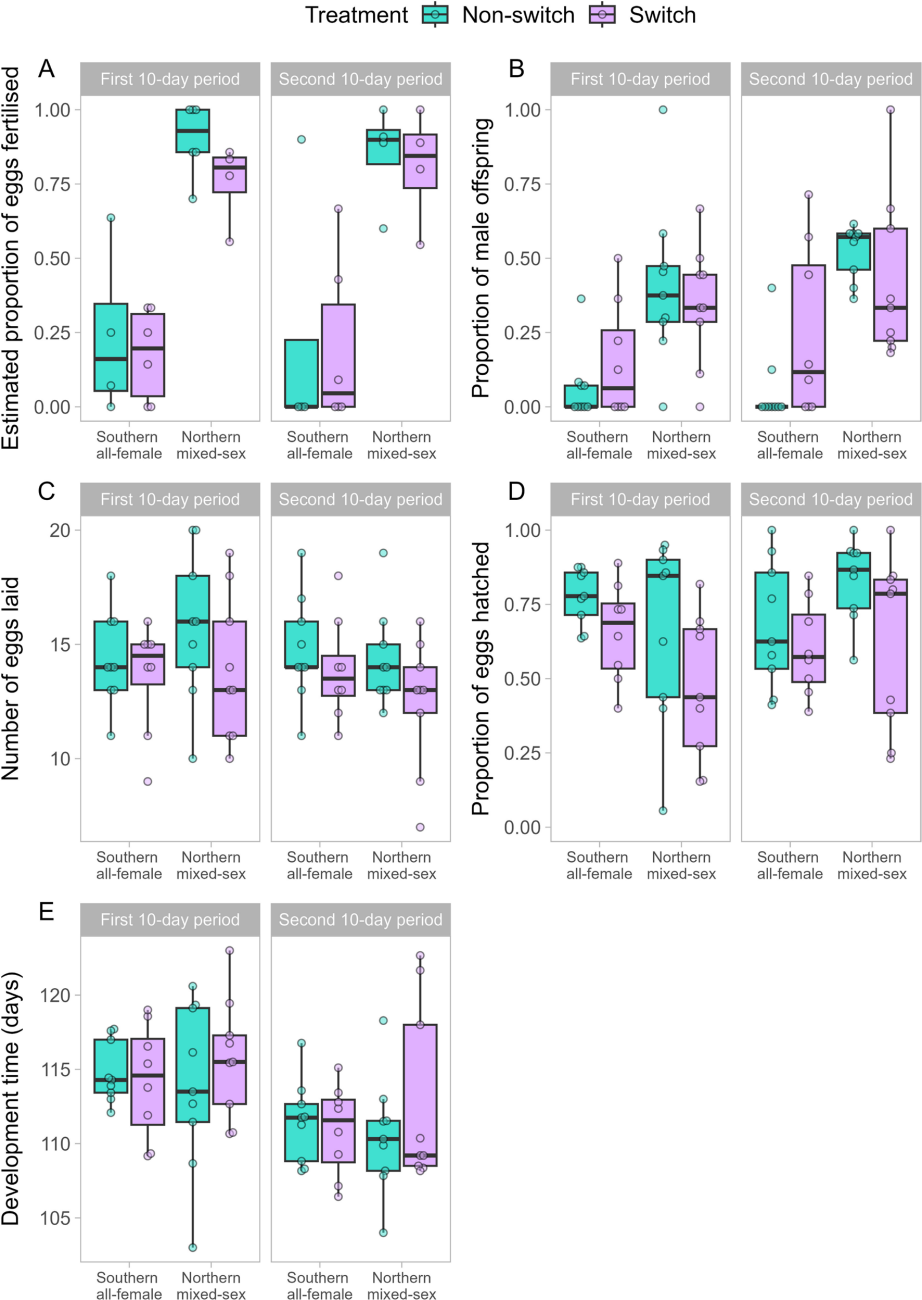
Fertilisation and reproductive performance. The non‐switch treatment groups are shown in turquoise/dark, whereas switch treatment groups are shown in lavender/light. Estimated proportion of eggs fertilised (A), proportion of male offspring (B), number of eggs laid (C), proportion of eggs that hatched (D) and egg development time (E) are shown on the *y*‐axis. Female population type (Southern all‐female vs. Northern mixed‐sex) is shown on the *x*‐axis. Panel columns represent egg collection period (left: first 10‐day period, right: second 10‐day period). Each point represents one brood × egg collection period combination. The black centre line inside each boxplot shows the median, whereas boxes show the inter‐quartile distance. The whiskers extend up to 1.5 times the inter‐quartile range.

On average, the estimated proportion of eggs fertilised across all mothers, treatment groups and egg collection periods was 53.4% (95% CI 36.7, 69.2). However, fertilisation rates were much lower for females from Southern all‐female populations (13.6%; 95% CI 5.57, 29.5) than for females from Northern mixed‐sex populations (89.2%; 95% CI 75.7, 95.6) across both the first and second 10‐day egg collection periods (Figure 3A, Table 1, Table S2). We did not detect any differences in fertilisation rate between first and second 10‐day egg collection periods, nor between switch and non‐switch treatment groups, nor any interactions among predictors.

**TABLE 1:** Output from a generalised linear mixed model with a binomial error distribution exploring the effects of treatment (non‐switch vs. switch), egg collection period (first vs. second 10‐day periods) and female population type (Southern all‐female [SAF] vs. Northern mixed‐sex [NMS]) on the estimated proportion of offspring that developed from fertilised eggs. Values in bold indicate significant effects (*p* < 0.05). CI stands for 95% confidence interval.

Predictors	Odds ratio	CI	*p*
Intercept	0.18	0.04–0.91	**0.039**
Treatment (switch)	0.76	0.09–6.19	0.801
Egg collection period (2nd 10‐day period)	0.78	0.21–2.86	0.704
Female population type (NMS)	**101.65**	**11.05–935.27**	**< 0.001**
Treatment (switch) × egg collection period (2nd 10‐day period)	1.65	0.30–9.18	0.569
Treatment (switch) × female population type (NMS)	0.26	0.01–5.07	0.374
Egg collection period (2nd 10‐day period) × female population type (NMS)	0.95	0.15–6.12	0.958
Treatment (switch) × egg collection period (2nd 10‐day period) × female population type (NMS)	1.2	0.10–14.02	0.887

### 3.2 Offspring Sex Ratio

**FIGURE 4:**
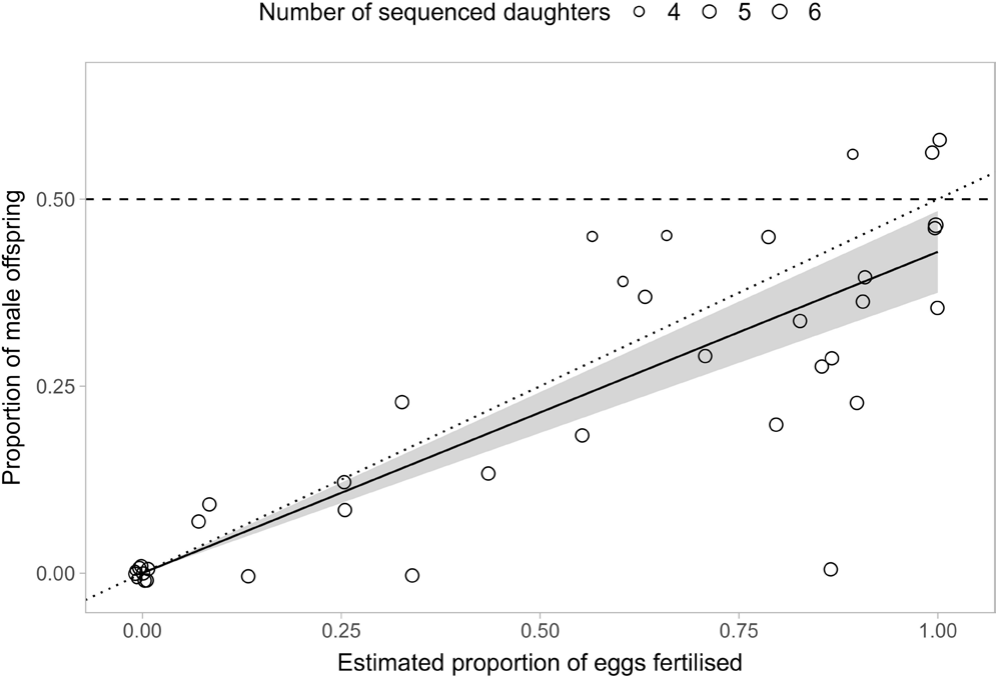
Relationship between the estimated proportion of eggs fertilised (*x*‐axis) and the proportion of male offspring (*y*‐axis). The dashed horizontal line represents an equal proportion of males and females in egg broods. The dotted line represents the expected relationship between the variables if fertilised eggs have an equal probability of developing into male versus female offspring, and the offspring sex ratio therefore reflects the fertilisation rate (*β* = 0.5). The solid line represents a linear regression where the intercept was forced to zero, with the 95% confidence interval for the slope shown as a shaded area (*β* = 0.429, 95% CI = 0.376–0.483, *R* ^2^ = 0.33).

As expected, the proportion of male offspring increased with fertilisation rate (Figure 4). Consequently, we found qualitatively identical results to those described for fertilisation rate: offspring sex ratio was not affected by treatment, egg collection period or their interaction (Table 2). However, similarly to fertilisation rate, we found that female population type affected offspring sex ratio: females descended from Southern all‐female populations produced 8.5% sons (95% CI 4.9, 14.4), whereas females descended from Northern mixed‐sex populations produced 40% sons (95% CI 30.5, 50) (Figure 3B, Table 2, Table S2).

**TABLE 2:** Output from a generalised linear mixed model with a binomial error distribution exploring the effects of treatment (non‐switch vs. switch), egg collection period (first vs. second 10‐day periods) and female population type (Southern all‐female [SAF] vs. Northern mixed‐sex [NMS]) on the proportion of male offspring. Values in bold indicate significant effects (*p* < 0.05). CI stands for 95% confidence interval.

Predictors	Odds ratios	CI	*p*
Intercept	0.05	0.02–0.14	**< 0.001**
Treatment (switch)	2.52	0.68–9.31	0.164
Egg collection period (2nd 10‐day period)	0.81	0.24–2.77	0.733
Female population type (NMS)	**11.00**	**3.33–36.32**	**< 0.001**
Treatment (switch) × egg collection period (2nd 10‐day period)	2.5	0.53–11.86	0.248
Treatment (switch) × female population type (NMS)	0.38	0.07–1.95	0.248
Egg collection period (2nd 10‐day period) × female population type (NMS)	2.42	0.62–9.54	0.206
Treatment (switch) × egg collection period (2nd 10‐day period) × female population type (NMS)	0.20	0.03–1.27	0.087

### 3.3 Fecundity

We found no evidence of effects of treatment, egg collection period or female population type on fecundity (number of eggs laid by each female; Figure 3C, Table 3).

**TABLE 3:** Output from a generalised linear mixed model with a Poisson error distribution exploring the effects of treatment (non‐switch vs. switch), egg collection period (first vs. second 10‐day periods) and female population type (Southern all‐female [SAF] vs. Northern mixed‐sex [NMS]) on fecundity (number of eggs laid). Values in bold indicate significant effects (*p* < 0.05). CI stands for 95% confidence interval.

Predictors	Incidence rate ratios	CI	*p*
Intercept	14.33	12.06–17.03	**< 0.001**
Treatment (switch)	0.95	0.74–1.23	0.697
Egg collection period (2nd 10‐day period)	1.03	0.81–1.31	0.805
Female population type (NMS)	1.1	0.87–1.40	0.43
Treatment (switch) × egg collection period (2nd 10‐day period)	0.99	0.69–1.41	0.946
Treatment (switch) × female population type (NMS)	0.93	0.65–1.31	0.667
Egg collection period (2nd 10‐day period) × female population type (NMS)	0.88	0.63–1.24	0.465
Treatment (switch) × egg collection period (2nd 10‐day period) × female population type (NMS)	1	0.61–1.65	0.995

### 3.4 Hatching Success

Overall, hatching success was 65.5%, with 649 hatchlings (*N*_daughters_ = 479; *N*_sons_ = 170) emerging from a total of 990 eggs laid. We found that the egg collection period interacted with female population type to affect hatching success (Figure 3D, Table 4). More specifically, the hatching success of eggs laid by females descended from Southern all‐female populations decreased by, on average, 9.5% from the first to the second 10‐day egg collection period, although this difference was not statistically significant in post hoc pairwise comparisons (*z* = 2.169, *p* = 0.132). In contrast, the hatching success of eggs laid by females descended from Northern mixed‐sex populations increased by, on average, 19% from the first to the second 10‐day egg collection period (Figure 3D, Table 4; *z* = 4.106, *p* < 0.001). Although we did not find statistical support for an effect of treatment, the data suggest a trend towards lower hatching success of eggs laid by females in the switch treatment group relative to the non‐switch treatment group (Figure 3D).

**TABLE 4:** Output from a generalised linear mixed model with a binomial error distribution exploring the effects of treatment (non‐switch vs. switch), egg collection period (first vs. second 10‐day periods) and female population type (Southern all‐female [SAF] vs. Northern mixed‐sex [NMS]) on hatching success. Values in bold indicate significant effects (*p* < 0.05). CI stands for 95% confidence interval.

Predictors	Odds ratios	CI	*p*
(Intercept)	3.94	2.13–7.27	**< 0.001**
Treatment (switch)	0.5	0.21–1.20	0.122
Egg collection period (2nd 10‐day period)	**0.54**	**0.30–0.94**	**0.031**
Female population type (NMS)	0.51	0.22–1.19	0.119
Treatment (switch) × egg collection period (2nd 10‐day period)	1.43	0.64–3.19	0.378
Treatment (switch) × female population type (NMS)	0.82	0.25–2.69	0.743
Egg collection period (2nd 10‐day period) × female population type (NMS)	**5.22**	**2.29–11.89**	**< 0.001**
Treatment (switch) × egg collection period (2nd 10‐day period) × female population type (NMS)	0.5	0.16–1.56	0.231

### 3.5 Egg Development Time

Overall, egg development time (days from first egg to first hatchling) in the non‐switch treatment group showed a similar pattern to that of the switch treatment group (Figure 3E, Table S2). We found no significant effects of treatment or female population type (Northern mixed‐sex vs. Southern all‐female) on egg development time, nor any significant interactions (Table 5). However, we observed a significant effect of the egg collection period (Figure 3E, Table 5). The mean development time for eggs laid during the first 10‐day period (115 days, 95% CI 114, 116) was ~4% greater than the mean development time for eggs laid during the second 10‐day period (111 days, 95% CI 110, 112) (Figure 3E, Table S2).

**TABLE 5:** Output from a linear mixed model with Gaussian distribution exploring the effects of treatment (non‐switch vs. switch), egg collection period (first vs. second 10‐day periods) and female population type (Southern all‐female [SAF] vs. Northern mixed‐sex [NMS]) on egg development time (days). Values in bold indicate significant effects (*p* < 0.05).

Predictors	Estimates	CI	*p*
(Intercept)	114.75	112.80 to 116.69	**< 0.001**
Treatment group (switch)	−0.53	−3.47 to 2.40	0.721
Female population type (NMS)	−0.27	−3.07 to 2.54	0.852
Timing of egg collection (2nd 10‐day period)	**−3.2**	**−5.21 to −1.19**	**0.002**
Treatment group (switch) × female population type (NMS)	1.78	−2.46 to 6.03	0.41
Treatment group (switch) × timing of egg collection (2nd 10‐day period)	−0.14	−3.23 to 2.95	0.928
Female population type (NMS) × timing of egg collection (2nd 10‐day period)	−0.61	−3.44 to 2.22	0.672
Treatment group (Switch) × female population type (NMS) × timing of egg collection (2nd 10‐day period)	−0.2	−4.64 to 4.24	0.929

### 3.6 Paternity

**FIGURE 5:**
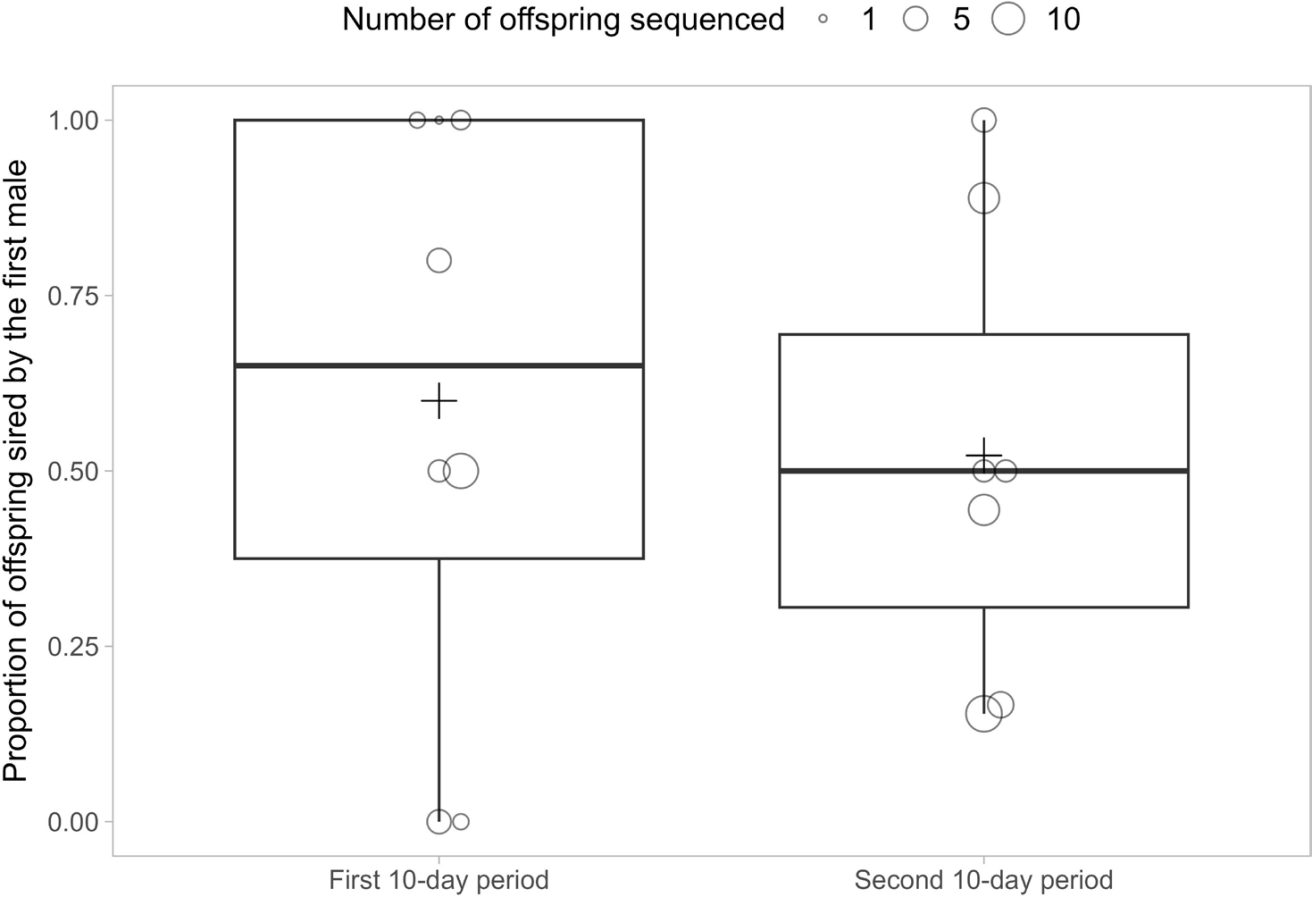
Proportion of sexually produced offspring sired by first male (*y*‐axis) during the first and second 10‐day periods (*x*‐axis). The centre black line inside each boxplot shows the median, and each box shows the inter‐quartile distance, and the whiskers show the range of values outside the inter‐quartile range. Plus signs represent the mean values. Each point represents one brood × egg collection period combination. Size of the points represent the number of sequenced offspring. We used offspring that developed from fertilised eggs (both sons and daughters) from the non‐switch treatment group only.

We found no clear pattern of sperm precedence: overall, the first and second male sired approximately equal proportions of offspring (Mann–Whitney *U* test: *W* = 17, *p* = 0.9438; Figure 5). The mean proportion of offspring sired by the first male was slightly higher during the first 10‐day period (60%) than during the second 10‐day period (52.2%), but we found no statistical support for an effect of egg collection period on the first male's fertilisation share (Figure 5, Figure S2, Table 6). There was also no statistical support for an effect of female population type. The first and second male's fertilisation share appeared to vary across families (Figure S2). The first male was the exclusive sire in two families, but the total number of offspring sequenced for these families was relatively small.

**TABLE 6:** Output from a generalised linear mixed model with a binomial error distribution exploring the effects of egg collection period (1st 10‐day vs. 2nd 10‐day periods) and female population type (Southern all‐female [SAF] vs. Northern mixed‐sex [NMS]) on the proportion of offspring sired by the first and second males in the non‐switch treatment group.

Predictors	Odds ratios	CI	*p*
(Intercept)	1.12	0.04–33.32	0.947
Egg collection period (2nd 10‐day period)	3.44	0.08–150.12	0.522
Female population type (NMS)	2.08	0.05–88.32	0.702
Egg collection period (2nd 10‐day period) × female population type (NMS)	0.13	0.00–7.54	0.328

## 4 Discussion

We found that switching reproductive modes did not affect the proportion of offspring produced from fertilised versus unfertilised eggs. *M. batesii* females that were initially unpaired (switch treatment) produced a similar proportion of their offspring from fertilised eggs in the first 10 days after being paired with a male, compared with females that had been paired for longer (non‐switch treatment). This means that females of this species can quickly switch reproductive modes and suggests that little or no change in oogenesis is required to transition from producing eggs that develop parthenogenetically (i.e., undergo automixis) to producing eggs that undergo fertilisation. We found no difference between population types in the rate of switching from parthenogenetic to fertilised eggs. However, females originating from Southern‐genotype all‐female populations mostly produced unfertilised eggs regardless of when they were paired with a male or when eggs were collected. Moreover, we did not observe any clear impacts from reproductive switching on reproductive performance. We also found that sperm mixing occurs in this facultatively parthenogenetic species, so that mating order does not affect male reproductive success, and females started using the sperm of the second male very quickly after male substitution.

The lack of effects of treatment, egg collection period or their interaction on both fertilisation rate (Figure 3A) and offspring sex ratio (Figure 3B) suggests that females can switch from laying parthenogenetic eggs to laying fertilised eggs almost immediately after mating. Since *M. batesii* eggs take several days to fully develop (Vasconcelos et al. 2025), our results suggest that switching from laying parthenogenetic eggs to laying fertilised eggs does not require any change in the structure of the eggs themselves. This implies the existence of a cytological mechanism that flexibly switches from fusion of the female pronucleus with the male pronucleus if fertilisation occurs to fusion of the female pronucleus with the nucleus of another female meiotic product in the absence of fertilisation. However, consistent with a previous study on this species (Wilner, Boldbaatar, et al. 2025), we found a highly female‐biased offspring sex ratio resulting from a low fertilisation rate among offspring produced by females originating from Southern all‐female populations, whereas females originating from Northern mixed‐sex populations produced near‐even offspring sex ratios resulting from a high fertilisation rate. Previous work showed that females in Southern all‐female populations are resistant to mating and fertilisation as a result of an evolved genotypic effect and a maternal effect associated with development from an unfertilised egg (‘impaternity’) (Wilner, Boldbaatar, et al. 2025). Southern all‐female populations appear to have been established for a long time and have been reproductively isolated from Northern mixed‐sex populations for many generations (Miller et al. 2024). In our study, most switch‐treatment females produced at least one son and/or one daughter from fertilised eggs, confirming that mating had occurred. Only one brood family (*N* = 22 offspring) contained only daughters and none of the sequenced daughters (*N* = 10) were from fertilised eggs. It is very likely that mating occurred in this brood family, as the pair was kept in the container over 20 days, minimising the likelihood of female escape from the male. However, it is possible that the male produced non‐viable sperm. Despite their low overall fertilisation rate, we found no evidence that females from Southern all‐female populations took longer to switch from laying exclusively parthenogenetic eggs to laying some fertilised eggs. This suggests that, despite some differences in reproductive biology of Southern all‐female vs. Northern mixed‐sex females, the processes involved in switching from parthenogenetic to sexual reproduction are similar in both.

We did not find clear evidence that switching from asexual reproduction (parthenogenesis) to sex has negative effects on reproductive performance for *M. batesii* females. Fecundity did not differ between switch and non‐switch treatment groups (Figure 3C). Our data suggest a trend towards lower egg hatching success in females that switched from parthenogenesis to sex across both population types and egg collection periods (Figure 3D), but this trend lacks statistical support. Nonetheless, this trend suggests a potential cost of switching that should be investigated in future experiments with a larger sample size. Costs of switching from parthenogenesis to sex appear to vary among facultatively parthenogenetic species. Potential costs of switching from parthenogenesis to sex were reported in the stick insect *E. tiaratum* (Burke et al. 2015), but no evidence of such costs was found in the stick insect *Sipyloidea laryyi* (Burke and Bonduriansky 2018a).

We observed an interaction of egg collection period and female population type on egg hatching success: females descended from Northern mixed‐sex populations laid more viable eggs during the second 10‐day period than during the first 10‐day period, while hatching success for females descended from Southern all‐female populations either remained stable or decreased over the course of the experiment (Figure 3D). It is not clear whether this effect of population type on egg hatching success resulted from genotypic differences or reproductive mode differences between the Southern all‐female and Northern mixed‐sex populations. Reproductive performance of Northern mixed‐sex females may have improved over the course of the experiment if they require several weeks after maturing to reach the peak of their performance. Age‐related changes in reproductive rate occur in many animals, although offspring viability tends to deteriorate as females age (Al‐Lawati and Bienefeld 2009; Fox 1993; Ivimey‐Cook and Moorad 2020). However, it remains unclear why females from Southern all‐female populations do not exhibit the same hatching success pattern across time as females from Northern mixed‐sex populations. Another possibility is that there is a distinct influence of male seminal fluid on egg viability, as work on other insect species shows that seminal fluid can reduce the egg hatching success temporarily (Prout and Clark 2000). Elevated mating rate soon after male introduction or replacement might thus have resulted in less viable eggs from the first 10‐day egg collection period. Perhaps females from Southern all‐female populations mated less frequently at the start of the experiment than did females from Northern mixed‐sex populations. We also observed that development time was shorter for eggs laid during the second 10‐day period compared with those laid during the first 10‐day period (Figure 3E). These changes could have occurred due to the effect of female ageing (Smith and Bohren 1975).

Lastly, we found no evidence of a clear sperm precedence pattern in *M. batesii* , as offspring collected during both the first and second 10‐day periods were sired at approximately equal rates by the first and second male (Figure 5). This suggests that relatively even sperm mixing occurs when an *M. batesii* female mates with multiple males. Sperm mixing has been observed in many other species, including pseudoscorpions (Zeh and Zeh 1994) and the obligately sexual stick insect *Timema cristinae* (Arbuthnott et al. 2015). Sperm mixing can result from the shape and function of reproductive organs of males and/or females. For example, the spermathecae (i.e., female sperm storage organs) may become pressured when filled with large amounts of sperm, which can lead to sperm mixing (Birkhead and Møller 1993; Cook et al. 1997; Zeh and Zeh 1994). Spermatheca shape can also play an important role in determining sperm precedence patterns, as certain shapes allow a great number of sperm to be stored for long‐term use (Schlager 1960; Walker 1980). The spermathecae in *M. batesii* range in shape from a plump bean‐like shape to sickle shaped (D. Wilner, unpublished data). It is possible that this spermatheca shape facilitates sperm mixing (see Bartlett et al. 1968; Walker 1980). Moreover, the relative amounts of sperm stored in the spermathecae need not reflect paternity share if females can exert control over paternity by selectively using sperm from different males (Birkhead and Møller 1993; Eberhard 1991; Pascini and Martins 2017). *M. batesii* females might benefit by distributing paternity evenly among the males that they had mated with as a strategy to reduce the likelihood of producing offspring sired by their own brothers, thereby avoiding inbreeding (Walker 1980). This could be important in *M. batesii* because neither sex can fly and most known populations are small (Miller et al. 2024), increasing the probability of full‐sib mating. Although some *M. batesii* populations persist entirely without males despite the near‐complete loss of heterozygosity and greatly reduced genetic variation resulting from parthenogenetic reproduction (Miller et al. 2024), females in mixed‐sex populations (where reproduction is typically sexual) might still benefit from outbreeding and production of high‐heterozygosity offspring. While we found near‐even sperm mixing in our experiment, it is possible that the sperm of the last male to copulate with a female might gradually displace the sperm of previous males in a longer timeframe than the one we tested here (20 days). Indeed, our data suggest a gradual reduction in the first male's paternity share over time, although this pattern was not supported statistically (Figure 5). Sperm mixing with or without eventual sperm displacement would explain why *M. batesii* males guard females for extended time periods (Boldbaatar et al. 2024).

Although paternity share was near‐evenly split between the first and second males on average, we also observed substantial variation in paternity share among families. Such variation could result from differences between the individual males in fertilisation success. Ejaculate traits, such as sperm length, velocity or quantity, can influence paternity (Boschetto et al. 2011; Decanini et al. 2013), albeit the potential of these traits to do so varies across species (Macartney et al. 2024). Genetic compatibility can also result in substantial variation in fertilisation success among males that mate with the same female, as observed in *Timema cristinae* (Arbuthnott et al. 2015). Our data do not allow us to determine which of these processes, if any, contributed to the variation that we observed in first versus second male siring success across families.

In summary, our results show that females can switch rapidly from laying unfertilised eggs to laying fertilised eggs, suggesting that this switch from parthenogenesis to sexual reproduction does not require a change in oogenesis. However, further research might reveal subtle changes in oogenesis or egg provisioning in response to reproductive mode switching. We also found near‐complete sperm mixing, but further research is required to address whether sperm from the previous male is eventually displaced by rival males. Likewise, future studies could usefully investigate whether sperm degrade rapidly within the spermatheca or if females actively seek to replace older sperm with new sperm. It would also be interesting to determine whether spermatheca morphology differs between females from Southern all‐female and Northern mixed‐sex populations. Sexual traits may be less functional in females descended from all‐female populations, where reproductive mode is exclusively asexual due to the absence of males (see Schwander et al. 2013).

## Author Contributions

**Jigmidmaa Boldbaatar:** conceptualization (equal), data curation (equal), formal analysis (equal), investigation (equal), methodology (equal), project administration (equal), software (equal), writing – original draft (equal), writing – review and editing (equal). **Pietro Pollo:** formal analysis (supporting), writing – review and editing (supporting). **Daniela Wilner:** conceptualization (supporting), methodology (supporting), writing – review and editing (supporting). **Nathan W. Burke:** conceptualization (supporting), methodology (supporting), writing – review and editing (supporting). **Russell Bonduriansky:** conceptualization (supporting), data curation (supporting), formal analysis (supporting), funding acquisition (lead), methodology (supporting), project administration (supporting), supervision (lead), writing – review and editing (supporting).

## Ethics Statement

The authors have nothing to report.

## Conflicts of Interest

The authors declare no conflicts of interest.

## Supporting information


Figure S1.

Figure S2.

Figure S3.

Tables S1–S2.

## Data Availability

All data and code used in this study are available at: https://doi.org/10.5061/dryad.5hqbzkhhd.
