# Supplementary figures and images for: Reproductive Transitions and Sperm Utilisation in a Facultatively Parthenogenetic Stick Insect
Source: Ecol Evol. 2025 Jul 7;15(7):e71766. doi: 10.1002/ece3.71766 (PMC12234150; doi:10.1002/ece3.71766)

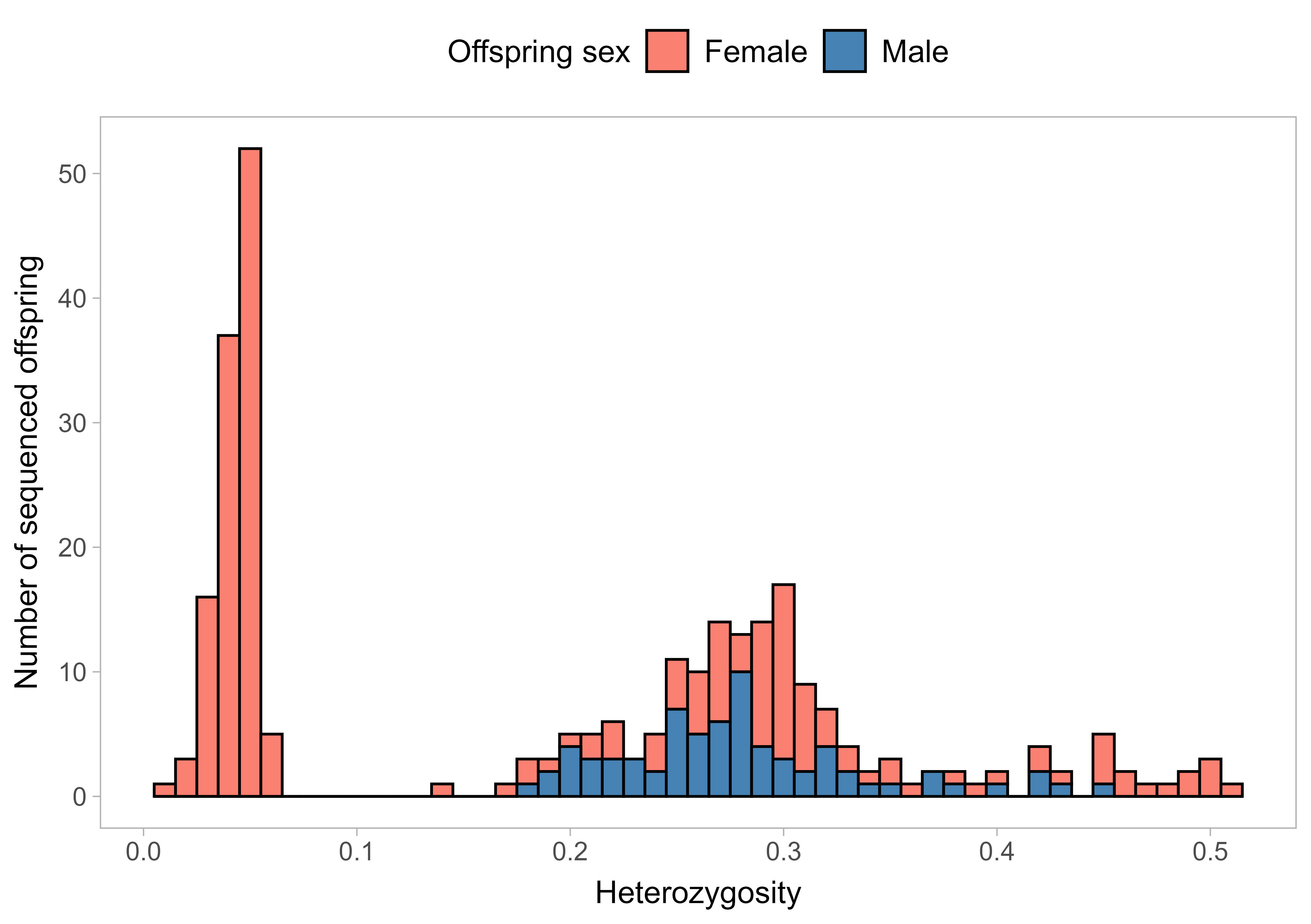

Supplement: Supplementary file 1 — Figure S1. [file ECE3-15-e71766-s001.tif]

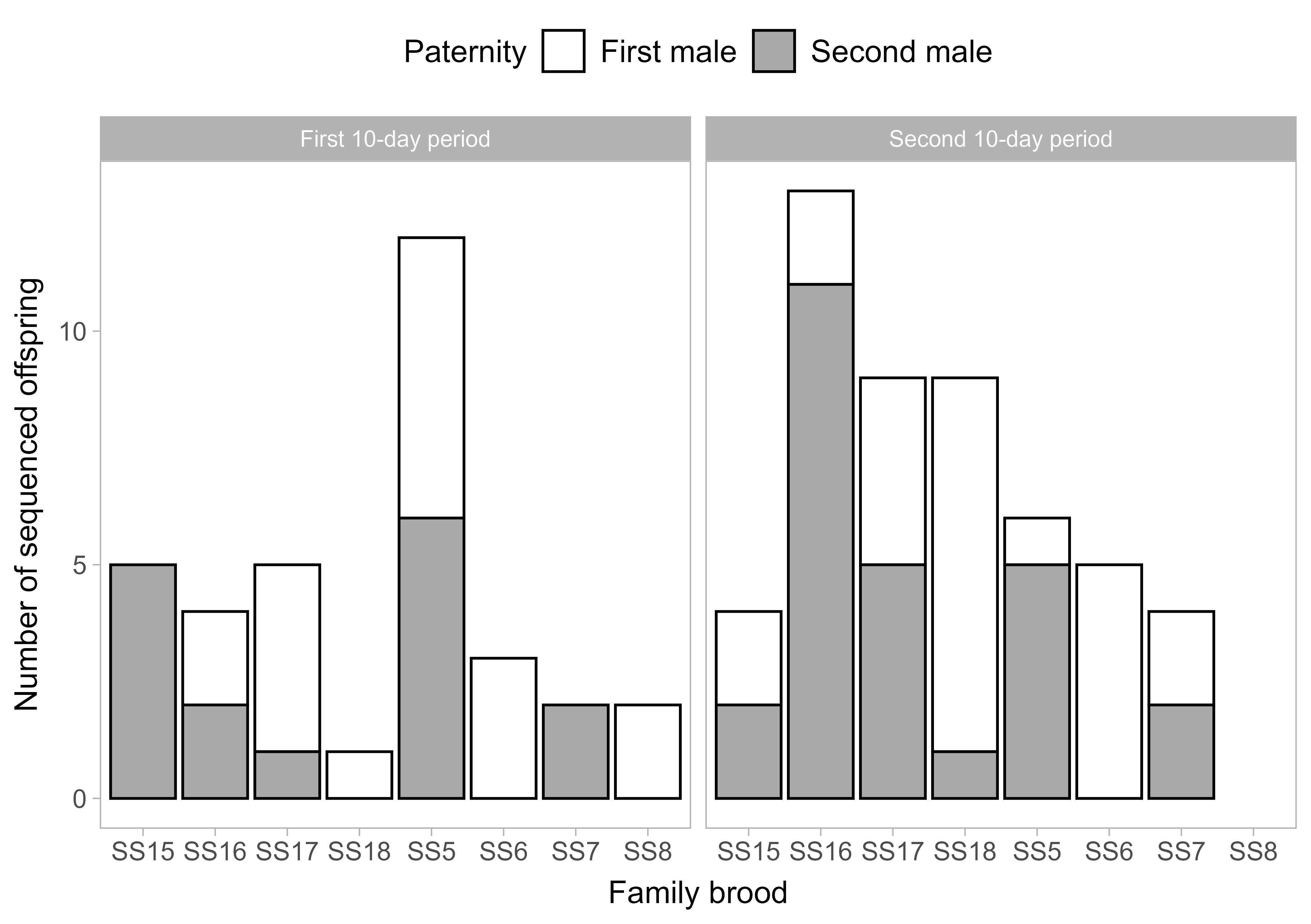

Supplement: Supplementary file 2 — Figure S2. [file ECE3-15-e71766-s003.tif]

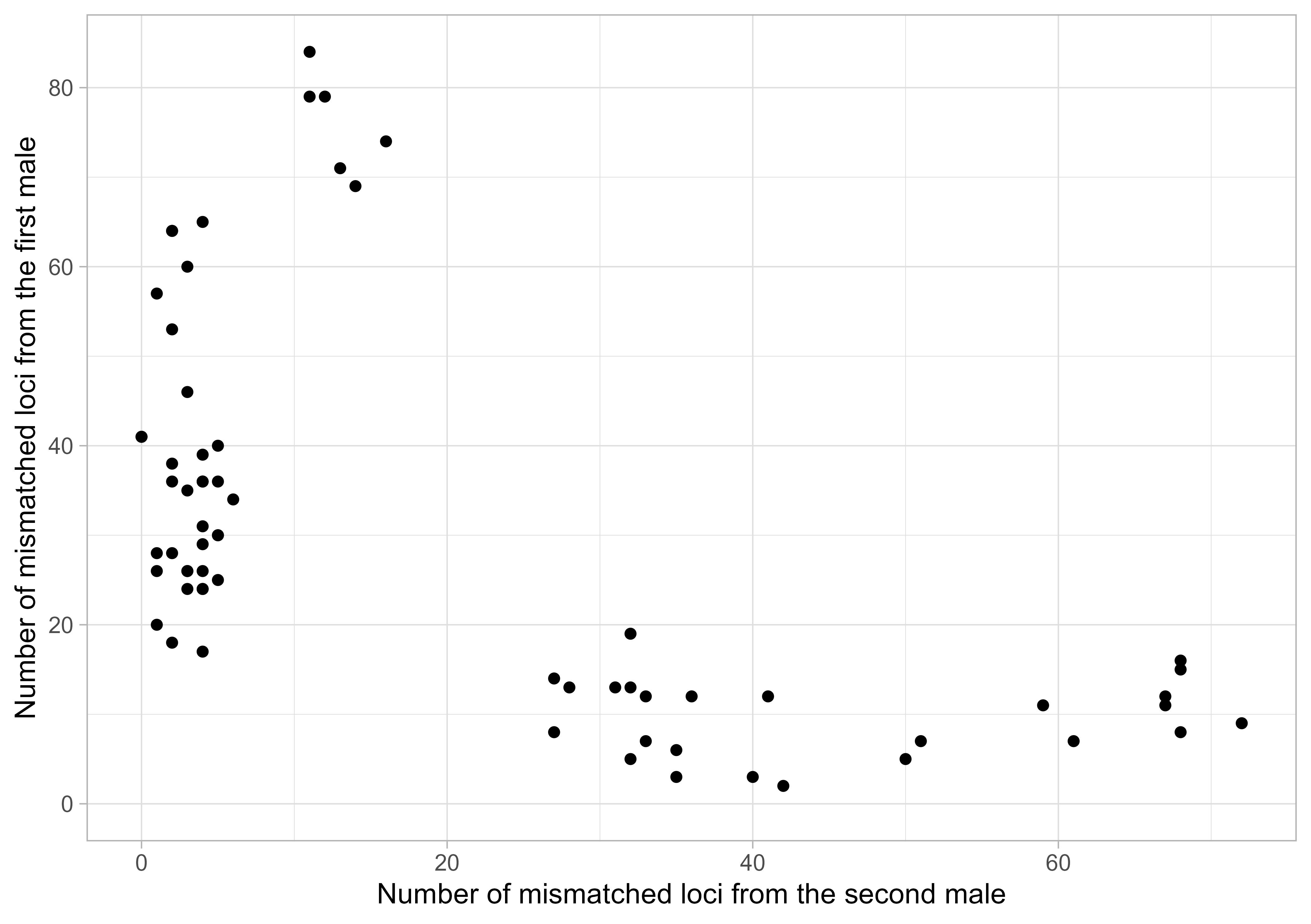

Supplement: Supplementary file 3 — Figure S3. [file ECE3-15-e71766-s004.tif]
