# Supplementary material for: Reproductive Transitions and Sperm Utilisation in a Facultatively Parthenogenetic Stick Insect
Source: Ecol Evol. 2025 Jul 7;15(7):e71766. doi: 10.1002/ece3.71766 (PMC12234150; doi:10.1002/ece3.71766)
Supplement: Supplementary file 4 — Tables S1–S2. [file ECE3-15-e71766-s002.docx]

**SUPPLEMENTARY MATERIALS**

**Table S1.** Linear mixed full model output with Gaussian distribution of hatchling development time (days). Treatment, hatchling sex, egg collection period and female population type were modelled as fixed effects and female ID was modelled as a random effect. Values in bold indicate significant effects (*p*< 0.05).

| *Predictors* | *Estimates* | *CI* | *p* |
| --- | --- | --- | --- |
| (Intercept) | 114.86 | 112.92 – 116.79 | **<0.001** |
| Treatment group (Switch) | -0.39 | -3.37 – 2.58 | 0.795 |
| Hatchling sex (male) | -1.75 | -7.26 – 3.76 | 0.534 |
| Female population type (NMS) | 0.45 | -2.52 – 3.43 | 0.765 |
| Timing of egg collection (2nd 10-day period) | -2.93 | -5.00 – -0.85 | **0.006** |
| Treatment group (Switch) * Hatchling sex (male) | 0.2 | -7.12 – 7.53 | 0.956 |
| Treatment group (Switch) * Female population type (NMS) | -0.6 | -5.16 – 3.97 | 0.798 |
| Hatchling sex (male) * Female population type (NMS) | -0.51 | -6.78 – 5.76 | 0.873 |
| Treatment group (Switch)* Timing of egg collection (2nd 10-day period) | 0.04 | -3.28 – 3.36 | 0.981 |
| Hatchling sex (male) * Timing of egg collection (2nd 10-day period) | -4.88 | -13.14 – 3.37 | 0.246 |
| Female population type (NMS) * Timing of egg collection (2nd 10-day period) | -1.92 | -5.30 – 1.46 | 0.265 |
| Treatment group (Switch)* Hatchling sex (male) * Female population type (NMS) | 6.04 | -2.74 – 14.82 | 0.177 |
| Treatment group (Switch)* Hatchling sex (male) * Timing of egg collection (2nd 10-day period) | 3.45 | -6.87 – 13.78 | 0.512 |
| Treatment group (Switch)* Female population type (NMS) * Timing of egg collection (2nd 10-day period) | 1.64 | -3.61 – 6.88 | 0.54 |
| Hatchling sex (male) * Female population type (NMS) * Timing of egg collection (2nd 10-day period) | 7.53 | -1.65 – 16.71 | 0.108 |
| Treatment group (Switch)* Hatchling sex (male) * Female population type (NMS) * Timing of egg collection (2nd 10-day period) | -8.97 | -21.21 – 3.28 | 0.151 |

**Table S2.** Estimated proportion of eggs fertilised, proportion of male offspring, and egg development time for each treatment group, egg collection period, and female population type. *N* stands for the number of females tested that produced the analysed offspring, while *SE* stands for standard error.

| **Treatment group** | **Egg collection period** | **Female population type** | **Estimated proportion of eggs fertilised** | | | **Proportion of male offspring** | | | **Egg development time (days)** | | |
| --- | --- | --- | --- | --- | --- | --- | --- | --- | --- | --- | --- |
|  |  |  | *N* | *Mean* | *SE* | *N* | *Mean* | *SE* | *N* | *Mean* | *SE* |
| Non-switch | 1st 10-day period | Southern all-female | 4 | 0.153 | 0.107 | 9.00 | 0.0496 | 0.0242 | 9.00 | 115 | 0.99 |
| Non-switch | 1st 10-day period | Northern mixed-sex | 6 | 0.948 | 0.0366 | 9.00 | 0.365 | 0.0764 | 9.00 | 114 | 1.03 |
| Non-switch | 2nd 10-day period | Southern all-female | 4 | 0.123 | 0.0915 | 9.00 | 0.0404 | 0.0222 | 9.00 | 112 | 1.03 |
| Non-switch | 2nd 10-day period | Northern mixed-sex | 4 | 0.931 | 0.051 | 9.00 | 0.529 | 0.0769 | 9.00 | 111 | 0.976 |
| Switch | 1st 10-day period | Southern all-female | 6 | 0.121 | 0.0733 | 8.00 | 0.116 | 0.0471 | 8.00 | 114 | 1.12 |
| Switch | 1st 10-day period | Northern mixed-sex | 4 | 0.785 | 0.13 | 9.00 | 0.357 | 0.0867 | 9.00 | 116 | 1.18 |
| Switch | 2nd 10-day period | Southern all-female | 6 | 0.15 | 0.0861 | 8.00 | 0.21 | 0.0679 | 8.00 | 111 | 1.14 |
| Switch | 2nd 10-day period | Northern mixed-sex | 4 | 0.841 | 0.104 | 9.00 | 0.353 | 0.0826 | 9.00 | 112 | 1.12 |

**Figure S1.** Frequency distribution of offspring heterozygosity where each individual’s heterozygosity percentage (x-axis) was calculated from both switch and non-switch treatments. The y-axis shows the number of offspring within each heterozygosity range. The daughters are represented in orange (light) whereas sons are represented in blue (dark).

**Figure S2**. The number of offspring sired by first male and second male (y-axis) during the first and second 10-day egg collection periods in non-switch family broods (x-axis). The number of offspring sired by the first male is shown in white while the number of offspring sired by the second male is shown in grey. None of the sequenced offspring of female SS8 from the second 10-day period were derived from fertilised eggs, and paternity therefore could not be determined for these offspring.

**Figure S3**. The number of mismatched loci from the first male (y-axis) and the second male (x-axis). Male loci were designated as mismatched if the male genotype crossed with the genotype of the known mother could not have produced the offspring genotype. The male with the fewest mismatched loci (of the 260 loci for which we obtained high-quality sequences) was identified as the sire. Note that the number of mismatched loci is always > 0 for both putative sires as a result of genotype call errors in the DNA sequence data.
